# Efficient Confirmation of Plant Viral Proteins and Identification of Specific Viral Strains by nanoLC-ESI-Q-TOF Using Single-Leaf-Tissue Samples

**DOI:** 10.3390/pathogens9110966

**Published:** 2020-11-19

**Authors:** Pavel Cejnar, Štěpánka Kučková, Jiří Šantrůček, Miroslav Glasa, Petr Komínek, Daniel Mihálik, Lucie Slavíková, Leona Leišová-Svobodová, Tatiana Smirnova, Radovan Hynek, Jiban Kumar Kundu, Pavel Ryšánek

**Affiliations:** 1 Department of Computing and Control Engineering, University of Chemistry and Technology in Prague, Technická 5, 166 28 Prague, Czech Republic; 2 Department of Plant Protection, Czech University of Life Sciences, Kamýcká 129, 165 00 Prague, Czech Republic; slavikova@vurv.cz (L.S.); rysanek@af.czu.cz (P.R.); 3 Department of Biochemistry and Microbiology, University of Chemistry and Technology in Prague, Technická 5, 166 28 Prague, Czech Republic; kuckovas@vscht.cz (Š.K.); santrucj@vscht.cz (J.Š.); smirnovt@vscht.cz (T.S.); hynekr@vscht.cz (R.H.); 4 Biomedical Research Center of the Slovak Academy of Sciences, Institute of Virology, Dúbravská cesta 9, 845 05 Bratislava, Slovakia; Miroslav.Glasa@savba.sk; 5 Faculty of Natural Sciences, University of Ss. Cyril and Methodius in Trnava, Nám. J. Herdu 2, 917 01 Trnava, Slovakia; daniel.mihalik@nppc.sk; 6 Division of Crop Protection and Plant Health, Crop Research Institute, Drnovská 507, 161 06 Prague, Czech Republic; kominek@vurv.cz (P.K.); leisova@vurv.cz (L.L.-S.); jiban@vurv.cz (J.K.K.); 7 National Agricultural and Food Centre, Research Institute of Plant Production, 921 68 Piešťany, Slovakia

**Keywords:** protein extraction protocol, LC-MS/MS, virus detection, viral proteins detection

## Abstract

Plant viruses are important pathogens that cause significant crop losses. A plant protein extraction protocol that combines crushing the tissue by a pestle in liquid nitrogen with subsequent crushing by a roller-ball crusher in urea solution, followed by RuBisCO depletion, reduction, alkylation, protein digestion, and ZipTip purification allowed us to substantially simplify the sample preparation by removing any other precipitation steps and to detect viral proteins from samples, even with less than 0.2 g of leaf tissue, by a medium resolution nanoLC-ESI-Q-TOF. The presence of capsid proteins or polyproteins of fourteen important viruses from seven different families (Geminiviridae, Luteoviridae, Bromoviridae, Caulimoviridae, Virgaviridae, Potyviridae, and Secoviridae) isolated from ten different economically important plant hosts was confirmed through many identified pathogen-specific peptides from a protein database of host proteins and potential pathogen proteins assembled separately for each host and based on existing online plant virus pathogen databases. The presented extraction protocol, combined with a medium resolution LC-MS/MS, represents a cost-efficient virus protein confirmation method that proved to be effective at identifying virus strains (as demonstrated for PPV, WDV) and distinct disease species of BYDV, as well as putative new viral protein sequences from single-plant-leaf tissue samples. Data are available via ProteomeXchange with identifier PXD022456.

## 1. Introduction

Plant viruses are important pathogens of many agricultural crops worldwide. Streaking epidemics of plant virus diseases have caused significant crop losses [1] with potential social impact [2,3]. New viruses or divergent viral strains and isolates have frequently been identified in recent years. Highly specific molecular detection techniques, like polymerase chain reaction (PCR), reverse transcription PCR (RT-PCR), quantitative PCR (qPCR), or loop-mediated isothermal amplification (LAMP), are focused on selected regions which could not be conserved enough among all strains, resulting in false-negative results. Similarly, serological methods, like enzyme-linked immunosorbent assays (ELISA), are often targeted to specific epitopes. In the case of new hosts infected by the existing pathogens, or even pathogens that have undergone evolutionary pressure, there is an increased need for alternative cost-effective detection techniques to provide tools for independent confirmation of the presence of virus pathogens.

The liquid chromatography-tandem mass spectrometry (LC-MS/MS) technique is well established as a wide-screen protein identification technique. However, in comparison to other wide-screen identification techniques like nucleic acids next-generation sequencing [4,5], its detection threshold, given by sufficient MS/MS protein fragment identification required, is high. When applied to the widely used *Escherichia coli* bacteria samples or HeLa human cell samples, the medium resolution LC-MS/MS [6] based instruments can identify several hundred or about a thousand proteins [7,8]. After optimization of the protein extraction protocol or using the depletion of the most abundant proteins, the total count of identified proteins could be increased by another few hundred proteins. Changing the technology to a high resolution LC-MS/MS could result in several thousands of identified proteins [9]. Subsequently, when state-of-the-art sample coverage is needed, the fractionation of samples and a long LC column of LC-MS/MS lead to many thousands of identified proteins [10,11] in Orbitrap or a quadrupole—time of flight (Q-TOF) mass spectrometers.

Mass spectrometry techniques have been used successfully for the detection of viral proteins [12,13], and especially for plant viruses [14]. At first, an extraction of virion particles or pure viral proteins was used and their subsequent identification was carried out by matrix-assisted laser desorption/ionization—time of flight (MALDI-TOF) or electrospray ionization (ESI). Later, the detection of viral proteins in total protein extracts was described in studies of differential changes in healthy and infected plant proteome [15,16], based mostly on 2D gel electrophoresis accompanied by MALDI-TOF and later directly on LC-MS/MS [16,17]. However, the initial amount of sampled tissue is often mentioned as a whole plant or a mixture of materials obtained from several plants.

To optimize protein extraction protocols, attention was focused on the precipitation steps (trichloroacetic acid-acetone, formic acid-acetonitrile, etc. were often used). Such steps add another source of variability to the results, which could lead to the failure to identify important proteins [18]. To eliminate this negative influence, increased weight of sampled material is required. Even though there are precipitation techniques reaching up to 100% efficiency [19,20] for small weight leaf samples (approximately 50–200 mg), we developed an extraction protocol without a chemical precipitation step. For plant samples, the protein extraction protocol must handle the disruption of cell walls (freeze-thaw cycle, mechanical disruption in liquid nitrogen, the addition of detergent, denaturation by heating, mechanical crushing in extraction buffer, etc.), together with the inhibition of any protein degradation process (adding a chaotropic agent or protease inhibitors) [20,21]. This is often followed by a selected contaminant removal process [19,22]. For LC-MS analysis, these steps are followed by the reduction of disulfide bonds, alkylation of free cysteine residues, and enzyme digestion to fragment proteins into peptides for identification purposes [23,24]. The proper use of all of these techniques must avoid incompatibility of the added agents with any subsequent enzyme digestion, and thus, these additional compounds must be often removed either by precipitation, micropipette-tip solid phase extraction, or at least by dilution to compatible concentrations. Steps leading to any side-effect modifications [25,26] can also significantly decrease the number of identified peptides. To overcome the limited LC column total protein capacity anticipating identification of low abundant proteins, the samples could be fractionated; however, this significantly increases the amount of LC-MS/MS analyzed samples. If only one sample should be kept for analysis, the most abundant proteins unrelated to the study, like RuBisCO (Ribulose-1, 5-bisphosphate carboxylase/oxygenase) for plants, should be depleted to increase the coverage. Two efficient RuBisCO depletion methods are commonly used [27,28,29]: precipitation with protamine sulfate or with phytate in the presence of Ca^2+^ ions.

To successfully identify proteins in plant samples, mass spectrometry techniques compare the detected peaks of mass spectra with a database of in silico digested proteins potentially present in the samples [30,31]. Thus, for the identification of virus pathogens, a database of potentially present viral proteins should be assembled first. Host protein databases must also be used when in vivo infection or plant-microbe interactions are studied. However, with increasing numbers of virus or host protein sequences included, the false discovery rate (FDR), maintaining the credibility of identified proteins above random identification by chance, could eliminate many more identified sequences [32,33]. To keep the ratio of false-positive results low, only recognized peptides longer than any certain threshold are used for search, and here, the set of potential virus pathogens tested in each sample is limited to only those occurring at a given plant genus, using available online databases like Plant Viruses Online [34] or Descriptions of Plant Viruses [35,36]. For successful identification, two unique detected peptide fragments, together with their MS/MS fragmentation spectra matching the supposed sequence with an above-the-threshold score, are considered a confirmed presence for the tested protein, provided that FDR filtering techniques are also employed on both the peptide identification and the protein identification level [37,38].

**Figure 1:**
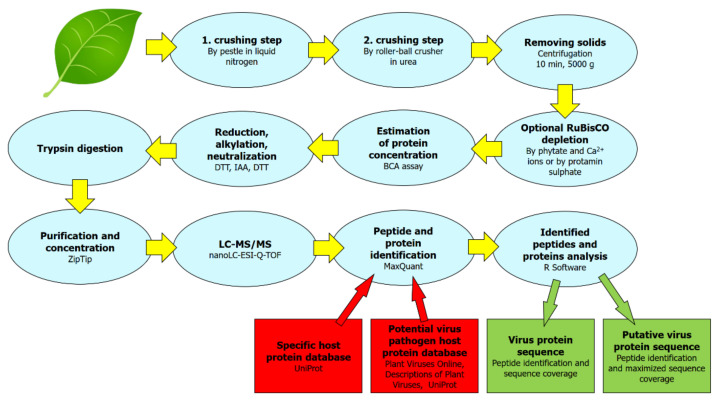
Sample processing scheme used in this work for efficient confirmation of viral proteins from plant leaf tissue.

**Figure 2:**
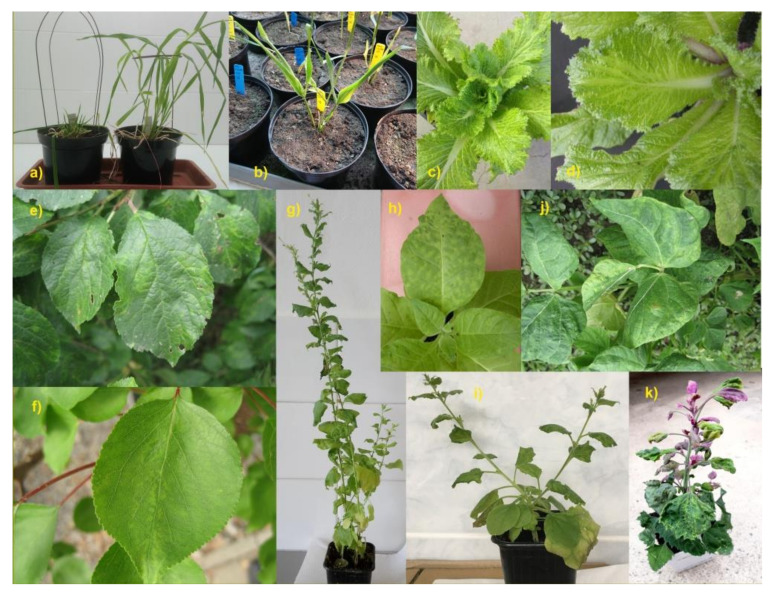
Virus-positive sampled plants: (**a**) barley cv. Doreen—plant with strong dwarfism (WDV) and a control, (**b**) wheat (WDV) cv. Fielder (**c**) Chinese cabbage (CaMV) (**d**) Chinese cabbage (TVCV) (**e**) plum (PPV) (**f**) apricot (PPV) (**g**) tobacco (PPV) (**h**) tobacco (TMV) (**i**) tobacco (TuMV) (**j**) common bean (BCMV) (**k**) goosefoot (BBWV-2).

In this work, we present an optimized plant protein extraction protocol (see Figure 1 for a scheme) enabling the extraction of host and virus proteins and subsequently confirming the presence of viral proteins, starting from as little as a single leaf of a plant with strong symptoms of infection. Such a protocol combined with at least medium resolution nanoLC-ESI-Q-TOF could be an efficient virus confirmation method for plants diagnosed by other low-threshold detection methods and left to develop strong symptoms. The small amount required for a protocol sample means that the plant need not be sacrificed, and can subsequently be used for further experiments. We tested the suitability of the protocol on many different plant species: economically important hosts, both monocots and dicots, such as barley (*Hordeum vulgare*), wheat (*Triticum aestivum*), Chinese cabbage (*Brassica rapa* subsp*. pekinensis*), tobacco (*Nicotiana tabacum*, *Nicotiana benthamiana*), plum (*Prunus domestica*), apricot (*Prunus armeniaca*), common bean (*Phaseolus vulgaris*), goosefoot (*Chenopodium amaranticolor*), sorghum (*Sorghum bicolor*), and maize (*Zea mays*). Each tested sample was experimentally inoculated by a virus, and the range of viruses tested includes both the DNA and RNA viruses (seven different virus families): Wheat dwarf virus (WDV) from Geminiviridae, Barley yellow dwarf virus (BYDV/BYDV-PAV) from Luteoviridae, Brome mosaic virus (BMV) and Tomato aspermy virus (TAV) from Bromoviridae, Cauliflower mosaic virus (CaMV) from Caulimoviridae, Tobacco mosaic virus (TMV) and Turnip vein clearing virus (TVCV) from Virgaviridae, Plum pox virus (PPV), Turnip mosaic virus (TuMV), Bean common mosaic virus (BCMV), Sorghum mosaic virus (SrMV), Sugarcane mosaic virus (SCMV) all from Potyviridae, Tobacco ringspot virus (TRSV) and Broad Bean Wilt Virus 2 (BBWV-2) from Secoviridae, see also Figure 2 and Supplementary Table S1.

## 2. Results

### 2.1. Two Crushing Steps Improve Identification of Plant Proteins

**Figure 3:**
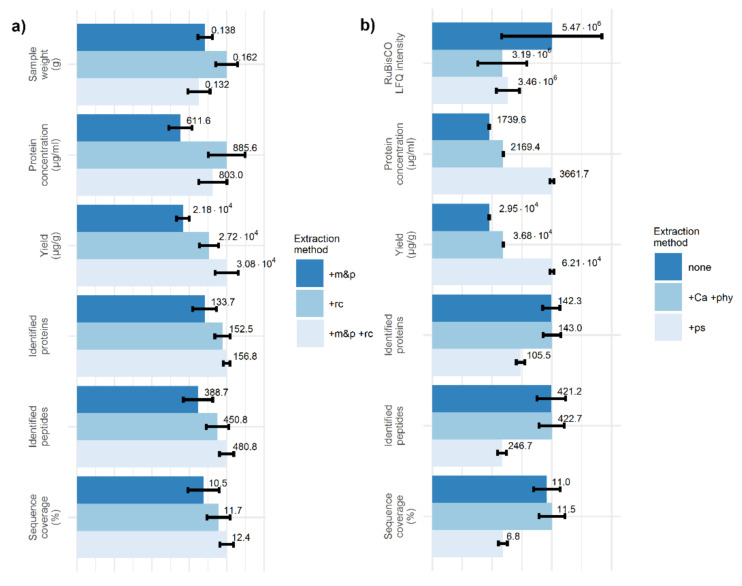
(**a**) Sample weights, the protein concentration of undiluted samples after the Removing solids step, protein yield per original leaf tissue weight used, identified peptides and proteins, and sample coverage for frozen winter wheat leaf tissue disrupted with a pestle in a mortar with liquid nitrogen (+m&p), or with a roller-ball crusher in urea solution (+rc), or by both (+m&p +rc). (**b**) MaxQuant LFQ normalized intensities for RuBisCO small chain, the protein concentration of undiluted samples after the Optional RuBisCO depletion step, protein yield per original leaf tissue weight used, identified peptides and proteins, and sample coverage for extraction protocol applied to frozen winter barley leaf tissue without RuBisCO depletion step (none), with a depletion step using phytate and Ca^2+^ ions (+Ca +phy), or with a depletion step using protamine sulfate (+ps). Error bars represent one standard error of the mean. All the groups contain exactly six samples (*n* = 6). For Identifed proteins, the total counts of MaxQuant proteinGroups, without contaminants, without proteins from reverse database, and without proteins only identified by site (by their peptide mass only, without supporting MS/MS spectrum) are listed.

To increase the protocol efficiency, two different crushing steps were included: crushing the material in liquid nitrogen by a pestle (crushing step 1, known from nucleic acids extraction methods) and crushing by a hand roller-ball crusher in a thick-walled plastic bag with a urea-based preservation solution (crushing step 2, where roller-ball crusher is often used in DAS-ELISA or similar methods). To confirm the efficiency of both steps, a reference material (plants of winter wheat, cv. Ludwig) was cultivated in the greenhouse and their leaves were cut by scissors to small pieces, put in one bag and stored at −80 °C. An extraction protocol (without any RuBisCO depletion steps) was applied either (1) only with crushing step 1, where the preservation solution from crushing step 2 was also applied, (2) only with crushing step 2, or (3) with both crushing steps, having six technical replicates per each group (18 in total). The samples were weighed after disruption in liquid nitrogen, just before adding the preservation solution. Figure 3a shows detailed characteristics of the extracted samples using UHPLC Dionex Ultimate 3000 RSLCnano connected to a mass spectrometer ESI-Q-TOF Bruker Maxis Impact for LC-MS/MS analysis. The disruption with a hand roller-ball crusher improves the number of identified peptides, number of identified proteins, and sample coverage, in comparison to disruption in liquid nitrogen only. For both crushing steps applied, there is another increase in these characteristics. The protein concentration of the extracted samples shows the highest correlation with the initial sample weight (Pearson’s correlation coefficient of 0.71), i.e., almost twice as high as with any other listed quantity. However, a significant increase in protein concentration can also be seen when both crushing steps are applied instead of only crushing step 1 (both groups having the same sample weight mean). To reduce the effect of the initial sample weight on the amount of extracted proteins, we can use the yield for subsequent comparisons; then, a significant increase is visible when both crushing steps are applied instead of only one.

In the second experiment, the effect of RuBisCO depletion steps was studied. Several plants of winter barley, cv. Doreen, cultivated in the greenhouse were cut to small pieces, stored at −80 °C, and the mixture was used as the reference material. Both crushing steps were applied and the solids were removed. Before optional depletion steps in the protocol, the supernatant of all the samples was merged to keep the same initial protein concentrations and then aliquoted to 18 Eppendorf tubes. For three groups (each having six technical replicates) the rest of the protocol, either (1) without RuBisCO depletion step (omitting the Optional RuBisCO depletion step), or (2) with RuBisCO depletion by phytate and Ca^2+^ ions (Optional RuBisCO depletion step, variant A), or (3) with RuBisCO depletion by protamine sulfate (Optional RuBisCO depletion step, variant B), was applied and the LC-MS/MS analysis was carried out as before. Figure 3b shows the mean and standard error of the mean for MaxQuant LFQ normalized intensities for RuBisCO small chain, the protein concentration of extracted samples after the Optional RuBisCO depletion step, the protein yield per original leaf tissue, identified peptides and proteins, and sample coverage among technical replicates. For estimation of RuBisCO intensity, a sum of MaxQuant label-free quantified (LFQ) intensities [30] for all detected peptides of Ribulose-1, 5-bisphosphate carboxylase/oxygenase small chain was used. For both depletion methods, there was an approximately 40% reduction in the recorded RuBisCO small chain LFQ intensity. The RuBisCO large subunit was also detected; however, a RuBisCO small chain was selected as a more sensitive marker, being less susceptible to cutting the extremely large peaks. It must be also noted that the MaxQuant protein intensity estimation algorithm attributes all the intensities of identified shared peptides (intensities of ‘razor peptides’) to the protein group with the largest number of total peptides identified, and thus, the proper intensity for pure RuBisCO small chain could also differ.

For depletion by protamine sulfate, a significant increase in both the yield and the protein concentration was visible; however, a reduced number of identified proteins, peptides, and reduced sequence coverage was also present in these samples. Protamine sulfate, a peptide-based compound, probably left some traces in the solution in which the BCA assay was carried out, leading to the overestimation of protein concentration. The reduced amount of leaf tissue proteins in the 1 µg extract injected to the LC column could result in reduced protein and peptide identification. For the other depletion step (phytate and Ca^2+^ ions), the increase due to the identification of low abundant proteins was very small using medium resolution LC-MS/MS, and could improve the detection rates probably only in combination with high resolution LC-MS/MS. If we want to analyze the consistent amount of proteins near the limit of the LC column, the protein concentration estimation step should not be omitted; thus, even though protamine sulfate could effectively reduce the RuBisCO levels, the interference with protein concentration estimation would be contra productive. For depletion of RuBisCO in our protocol, the usage of phytate and Ca^+^ ions is suggested.

### 2.2. Plant Virus Pathogen Capsid Proteins Could Be Efficiently Confirmed in Samples of Plants with Strong Infection Using the Double Crushing Extraction Protocol Followed by nanoLC-ESI-Q-TOF

For each cultivated host inoculated by a virus, the leaves were sampled and extracted (see Table 1 for a list of hosts and Supplementary File S1 for cultivation and inoculation conditions). The identified peptides in the samples were searched for pathogen-specific peptides. A list of potentially available virus pathogens for a given plant taxonomic genus was assembled using two available online databases of plant virus pathogens: Plant Viruses Online and Descriptions of Plant Viruses. Table 1 shows that the protocol is able to identify viral capsid proteins and other viral proteins in at least ten different plant hosts. In Supplementary File S2, all the identified viral proteins with the sequence isoform with the highest amino acid coverage, identified peptide fragments, and their protein alignments are listed. All identified peptides of viral proteins were then searched for occurrence in plant host protein sequences, and no such occurrence was found. Thus, the peptides identified in viral proteins were unique to identified virus sequences and could not be accidentally interpreted as identified virus sequences due to their incident colocation in the plant host proteome.

For the total sum of amino acids in a sequence covered by the identified fragments of all detected viral proteins of inoculated viruses, more than 100 amino acids were covered for each sample, with only one exception (the 06b/plum sample, 57 amino acid covered sequence); however, in many cases, this total sum reached several hundreds of amino acids (936 AA at most, for the 04a/tobacco sample). For the distinct viral proteins of both tested DNA viruses, the highest (for WDV, 147 AA of 260 AA or 56.5%) or second highest (for CaMV, 110 AA of 489 AA or 22.5%) amino acid sequence coverage was reported for their capsid proteins. For CaMV, the only protein with higher amino acid coverage was its translational activator (ORF6, 379 AA of 519 AA or 73.0%). For RNA viruses, a genome polyprotein is often expressed, which is subsequently digested to distinct viral proteins. If only final viral proteins instead of genome polyprotein are accounted for, then the highest amino acid sequence coverage was also reached for the capsid proteins. Similarly, if we compare the relative sequence coverage of viral proteins, the relative sequence coverage for capsid proteins was higher than for other detected viral proteins; it was at least 17.2% (for PPV capsid protein in the 06b/plum sample or 57 AA of 330 AA).

For mass spectrometry peptide fragment identification, only fragments equal or longer than the selected threshold are searched, and thus, in Table 1, a sequence coverage on ≥7 amino acid sequences is also reported, i.e., the relative coverage for detected fragments on protein sequence, from which all fragments shorter than a given threshold (seven amino acids) were removed. However, even on these reduced sequences, the relative coverage does not essentially differ (20.5% to 96.7%, median 43.9%), in comparison to values on protein sequences of the original length (17.2% to 77.8%, median 32.9%).

If the detected peptide fragments for a given virus protein cannot be aligned to only one protein sequence from the database, but can be aligned to other known sequence isoform, then these alignments to other protein isoforms are reported in MaxQuant as different protein groups for the studied protein. The frequent occurrence of different protein groups for the same viral protein lead as to the construction of putative protein sequence maximizing the sequence coverage by the detected peptides. Briefly, for each viral protein, an existing protein sequence with the highest detected sequence coverage was selected as an original sequence. All peptide fragments detected in the sample were extracted and locally aligned to the original protein sequence, keeping only the alignments with few modifications to the original sequence. Then, a putative sequence was constructed based on the original protein sequence and modifications included in the aligned peptides. The resulting amino acid coverage for these putative sequences is listed in Table 1. All the constructed putative viral protein sequences with detected peptide fragments and their alignment are reported in Supplementary File S3. The highest increase in sequence coverage can be seen for PPV in tobacco (from 936 AA to 1073 AA), SCMV in maize (from 343 AA to 398 AA), and BBWV-2 in goosefoot (from 275 AA to 327 AA). However, for some other viruses, even with high amino acid coverage in the capsid protein or polyprotein, no increase is obtained this way (WDV and BMV in cereal samples, TMV in tobacco).

Regarding potential misidentification, there is high sequence similarity of SrMV and SCMV, and thus, many peptides of SCMV were also detected in the sorghum sample. However, only one of them was an unique peptide for the SrMV sequence. Similarly, in the maize sample, several peptides of SrMV and SCMV shared sequence were also detected in the sample, but no unique peptide was detected, and thus, there is no entry in the related MaxQuant proteinGroups.txt search results file. For other samples, occasionally, a few peptides of other viruses can be also detected (e.g., see tobacco or goosefoot samples), but with, at most, one unique peptide. The other detected peptides are only those which are also shared with other viral sequences. These occasional occurrences are supposed to be a computer algorithm artifact (i.e., a fragment identified by chance due to a too large search protein database and not captured by FDR or target-decoy database search strategy), validating the requirement for at least two unique peptide fragments to be identified for sequence confirmation.

### 2.3. The nanoLC-ESI-Q-TOF Based Detection Method with Optimized Extraction Protocol Allows Discrimination of Virus Strains or Distinct Disease Species Based on Detected Fragments

For three different virus-caused diseases (WDV, PPV, and BYDV), several different virus strains (for WDV and PPV) or distinct diseases species (for BYDV, where sources of BYDV disease are classified as distinct virus species) are present in the Czech Republic. We focused on the classification of the detected disease source in the samples even to specific virus strains or, for BYDV, to the identification of BYDV distinct disease species.

**Figure 4:**
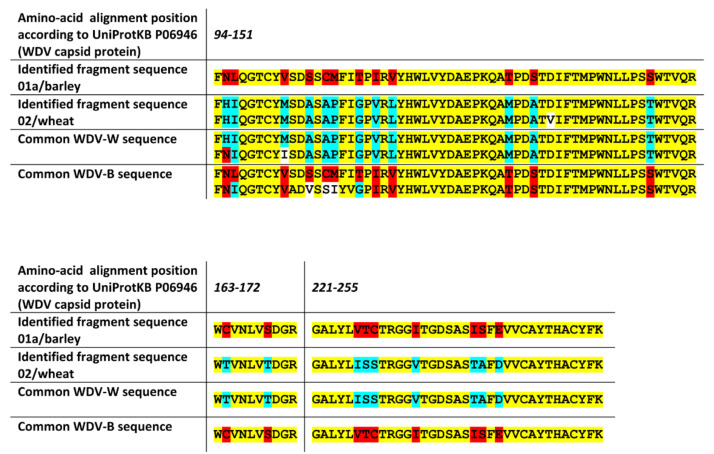
Alignment of detected discriminating peptide fragments of WDV capsid protein and strain-specific sequences for a given region in WDV-W and WDV-B strains. The positions with the same amino acid (yellow), with the detected amino acid in fragments similar to WDV-B (red), and with the detected amino acid in fragments similar to WDV-W (blue) are highlighted in different colors.

For WDV, where both WDV-Wheat (WDV-W) and WDV-Barley (WDV-B) strains occur in the Czech Republic, differences in many regions of the capsid protein amino acid sequence exist among these strains. Due to the high variability between WDV isolates of both strains, no single strain-specific sequence exists for many regions, and thus, several strain-specific sequences are often reported according to sequence subgroups. The high amino acid sequence coverage for tested samples and frequent strain-specific sequence differences allowed proper classification of the samples to be based here on many single amino acid differences (see Figure 4). The WDV strain of the barley sample was determined as WDV-B, and the WDV strain of the wheat sample was successfully determined to be WDV-W, which is in agreement with the used inoculation strain.

**Figure 5:**
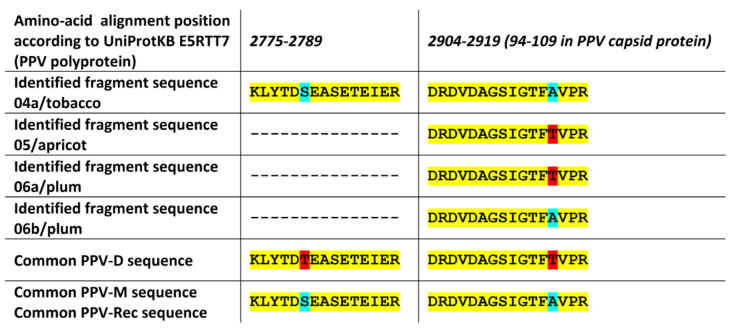
Alignment of detected discriminating peptide fragments of PPV polyprotein and strain-specific sequences for given regions in PPV-D and PPV-M strains. The positions with the same amino acid (yellow), with the detected amino acid in fragments similar to PPV-D (red), and with the detected amino acid in fragments similar to PPV-M (blue) are highlighted in different colors. PPV-Rec strain shares its amino acid sequence at the end of the genome polyprotein (including the capsid protein sequence) with the PPV-M strain.

In the case of PPV, PPV-D, and PPV-M strains, these occur in the Czech Republic and Slovakia, together with their recombined strain PPV-Rec (sharing the C-terminus of NIb and entire capsid protein amino acid sequence with PPV-M). Most of the identified fragments for all four PPV samples were detected in regions where strain-specific sequences are equal, and only one or two peptide fragments were detected and identified in regions where strain-specific sequence differs for tested strains (see Figure 5). Based on their identification, the 04a/tobacco sample can be determined to be infected with PPV-M strain or PPV-Rec, the 05/apricot and the 06a/plum as infected with PPV-D strain, and the 06b/plum as infected with PPV-M or PPV-Rec strain, which is in agreement with the strain identity of isolates used for inoculation of the tested plants.

**Figure 6:**
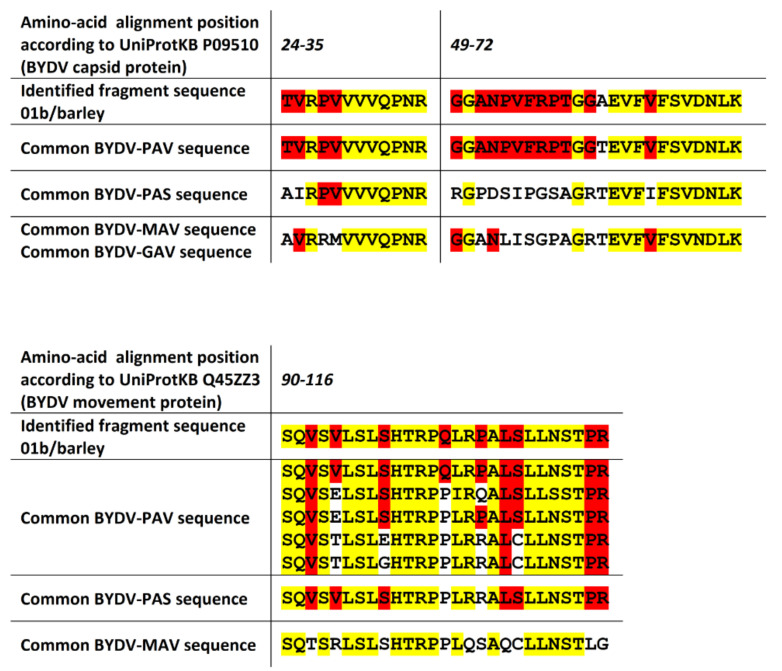
Alignment of detected discriminating peptide fragments of BYDV capsid and movement protein and strain-specific sequences for a given region in BYDV-PAS, BYDV-PAV, BYDV-MAV (and BYDV-GAV if the sequences are equal) strains. The positions with the same amino acid among all sequences (yellow) and positions with the same amino acids as in an identified fragment (red) highlighted by different colors.

Even though only two fragments of the capsid protein of BYDV were detected, these peptides are part of the region where the BYDV disease species occurring in the Czech Republic (see [40]) differ in their amino acid sequence. Both detected capsid-protein peptide fragments for BYDV confirmed the virus sample to be BYDV-PAV (see Figure 6), which is in agreement with the virus used for inoculation. Only one of the two detected movement-protein peptide fragments also allowed discrimination of the BYDV disease species for the detected virus. Even though the fragment is from a region where several species-specific sequences exist, this fragment also confirmed the virus sample to be BYDV-PAV.

## 3. Discussion

Although most methods described in this paper are generally used for proteomic analysis, the adaptation of the methods to identify viruses that may be present in low abundance is not a routine process. The presented protocol for protein extraction from leaf tissues employs techniques that are well known from nucleic acid protocol extractions—i.e., disruption of leaf samples in liquid nitrogen, the use of a chaotropic agent for protein denaturation, and techniques optimized for DAS-ELISA plant virus protein targeted methods—applying crushing by a roller-ball crusher in a preserving buffer. The high concentrations of the chaotropic agent, urea, are lowered by dilution to noninhibiting concentrations for trypsin digestion to avoid any incompatibility. The final concentration and desalting step are made using a micropipette-tip solid phase extraction, a technique which is similar to subsequent LC column protein separation. Such a protocol easily fulfills protein sample limits of LC columns, and subsequent nanoLC-ESI-Q-TOF identification makes it possible to confirm viral capsid proteins on a wide range of host plants, including grasses and flowering plants or trees. The number of identified peptides and high protein sequence coverage of viral capsid proteins even allowed us to discriminate specific virus strains or disease species based on the viral protein sequence analysis.

Even though the relative coverage of detected peptide fragments on ≥7 amino acid sequences in the samples reached 96.7% for BMV capsid protein on barley, for WDV capsid proteins, for example, this ratio stopped at 77.8%, on both highly infected barley and wheat samples. The identification of peptide fragments in recorded spectra is still based on the provided protein sequences and their modifications. Any missed modification in the setup of the database search could lead to missed identification of peptides, despite the fact that the peaks of these peptides are still present in the recorded spectra. Even if the proper modifications are included, for some of them, like phosphorylation, their detection by mass spectrometry techniques is generally complicated due to their different charge states and subsequent problems with ionization [41].

While the identification of peptides from MS/MS spectra is continuously improving [42,43], the significant reduction of the assembled protein database to be searched is an important factor for the elimination of false-negative results, where all these sequences are also used through control target-decoy database search strategy. For the detection of virus pathogens, a reduction to only a host and potentially available virus species is suggested. General online sources for potential plant host–virus interactions are currently not easily available. Frequently used sources, such as Plant Viruses Online or Descriptions of Plant Viruses, are reportedly not updated, even though they are still important sources of information. Selective information aggregated by plant hosts can be found for important agricultural crops and viruses [44,45]; however, a manual search in literature through protein database sources is required to obtain an up-to-date list of potential virus pathogens for a specific host, based on existing virus taxonomy [46].

Even though the absolute amino acid coverage and relative coverage of potentially misidentified sequences in the samples remain zero or very low (for unique detected viral protein fragments) or at least low (for all detected fragments including the shared ones) in comparison to detected coverages of proteins supposed for confirmation, the potential random identification of peptides of other viral sequences requires a relatively high amount of the protein to be present in the sample, resulting in high sequence coverage with several unique peptides identified. The advantage of the peptide-based identification method is its ability to reconstruct the protein sequence, provided that the sequence information is available in proteome databases. Even for the new viruses or mutated virus strains, if the amino acid sequences of their viral proteins share at least some parts with other related and already identified viruses, there is a high probability of identification of the part of the protein and confirmation of its presence. A putative sequence can be then reconstructed if there is an assumption for mutated virus strain or new virus present. However, a special caution must be made in case of co-infections. For distinct viruses with viral proteins sharing the large regions of the same protein sequence, many peptides could be identified from these regions. The decision of whether coinfection or infection by only a new species occurs will be then dependent on the identification of a few unique peptides from regions where the sequences should differ. A high sequence coverage could alleviate the issue, but generally, confirmation by another detection method would be then required, although the supporting method could be highly target-specific, like PCR.

The presented double crushing step extraction protocol combined with at least medium resolution LC-MS/MS allows confirmation of viral proteins even from single-leaf-tissue samples and can serve as an efficient viral protein confirmation method for highly infected samples.

## 4. Materials and Methods

### 4.1. Protein Sample Extraction and Preparation Protocol

Crushing step 1: Leaf tissue was cut and homogenized in liquid nitrogen, ground into a fine powder using mortar and pestle, and collected in an Eppendorf tube and weighted. Crushing step 2: The tissue from the previous step was transferred (or the remains were eluted by pipette) to a thick-walled plastic bag, and 5 mL of preservation solution (0.4 M ammonium hydrogen carbonate, 8 M urea, pH 7.8–8.0) was added. The content of the plastic bags was homogenized with a hand roller ball crusher homogenizer (Bioreba, Reinach, Switzerland, see Supplementary Figure S1) without removing the content from the bags. Removing solids: 1 mL of the liquid part was pipetted to an Eppendorf tube and centrifuged for 10 min at 5000× g to remove any remaining solids. The supernatant was pipetted to a new tube. Optional RuBisCO depletion step: Optionally, RuBisCO depletion procedure variant A (using phytate and Ca^2+^) or variant B (using protamine sulfate) can be subsequently included, or the extraction can continue directly to the next step (protein concentration estimation). Variant A: 200 μL of supernatant was pipetted to a new tube, and 20 μL of 100 mM CaCl_2_ (dissolved in 8 M urea) was added. Subsequently, 20 μL of 100 mM phytate (dissolved in 8M urea) was added. The sample was left incubated for 10 min in a shaker (42 °C, 500 rpm) and centrifuged for 15 min (16,100× g, room temperature). Variant B: 200 μL of supernatant was pipetted to a new tube, and 20 μL of 5% protamine sulfate (dissolved in 8M urea) was added. The sample was incubated for 30 min at 4 °C on ice and centrifuged for 15 min (12,000× g, room temperature). Protein concentration estimation: For each sample, an aliquot was diluted eight times by distilled deionized water and the concentration was estimated using the BCA assay (Thermo Fisher Pierce BCA Protein Assay Kit, Thermo Fisher Scientific, Waltham, MA, USA). Pipetting the same protein amount: For subsequent steps, the same protein amount (here 100 μg) from each extracted sample of its original concentration (not diluted as in the previous step) was pipetted to a new tube to proceed to subsequent steps in a convenient volume. Reduction step: Dithiothreitol (Sigma-Aldrich, St. Louis, MO, USA) was added to the final concentration of 5 mM, and the samples were left in a thermoblock for 30 min (60 °C, 300 rpm). Alkylation step: After cooling to room temperature, iodoacetamide (Sigma-Aldrich) was added to give a final concentration of 10 mM. The samples were then left for 30 min at room temperature in the dark. Neutralization of remains of iodoacetamide: Dithiothreitol was added to give a final concentration of 5 mM, and the samples were left for 30 min at room temperature. Trypsin digestion step: An aliquot of 2 μg of protein was diluted eight times by distilled deionized water, and 1 μL of trypsin (0.1 μg/μL fresh solution in distilled deionized water) was added. If required, the aliquot used for digestion can be lowered down to 1 µg of protein, and the dilution ratio can be also optionally lowered to four times for samples of low protein concentration, to keep the maximum volume of 20 µL for the reaction mixture in order to maintain the efficiency of the digestion and subsequent concentration. The reaction mixture was then incubated for 16 h (overnight) at 37 °C. Concentration step: The digestion was stopped by the addition of 10% trifluoroacetic acid (Sigma-Aldrich) to a final concentration of 0.5%, and the sample was concentrated using ZipTip (Millipore, Burlington, MA, USA) containing reverse phase C18 according to the supplied protocol with the elution solution of 5 µL of 50% acetonitrile and 0.1% TFA in deionized water. The tubes were then left open in air to evaporate the supernatant, leaving the solid extract.

### 4.2. LC-MS/MS

Mass spectrometry measurements were carried out using liquid chromatography column with nanoliter per min flow ranges (nanoLC), electrospray ionization (ESI), and quadrupole time-of-flight (Q-TOF) technology; i.e., here a UHPLC Dionex Ultimate 3000 RSLCnano (Dionex, Sunnyvale, CA, USA) was connected to a mass spectrometer ESI-Q-TOF Maxis Impact (Bruker, Billerica, MA, USA). The extracted sample was dissolved in the mixture of water:acetonitrile:formic acid (97:3:0.1%), and then 1 µg of peptides in the injection volume of 1 µL was loaded on a trap column, Acclaim PepMap 100 C18 (100 μm × 2 cm, particle size 5 μm, Dionex, Germany), with a mobile phase A (0.1% formic acid in water) flow rate of 5 μL/min for 5 min. The peptides were eluted from the trap column to the analytical column, Acclaim PepMap RSLC C18 (75 μm × 150 mm, particle size 2 μm), by mobile phase B (0.1% formic acid in acetonitrile) using the following gradient: 0–5 min 3% B, 5–95 min 3–35% B, 97 min 90% B, 97–110 90% B, 112 min 3% B, 112–120 min 3% B. The flow rate during gradient separation was set to 0.3 μL/min. The peptides were eluted directly to the ESI source—Captive spray (Bruker Daltonics, Germany). Measurements were performed in data-dependent analysis mode with precursor-ion selection in the range of 400–1400 *m/z*; an MS spectrum was recorded every 3 s and MS/MS spectra were collected at the speed of 4–16 Hz, depending on the intensity of the precursors. Dynamic exclusion was set to 1 min, preferred charge state to 2–5, and singly charged precursors were excluded. Collision induced dissociation MS/MS spectra were recorded in the range of 50–2200 *m/z* and profile spectra were saved. The mass spectrometry proteomics data and search results were deposited to the ProteomeXchange Consortium via the PRIDE partner repository [47] with the dataset identifier PXD022456.

### 4.3. Protein Identification and Protein Databases Searched

The peptides in raw spectra were identified and quantified by MaxQuant [11] 1.6.7.0 for Windows using reverse sequences for target-decoy database search strategy [48] and applying a 1% false discovery rate (FDR) for both the peptide spectrum match and protein group levels. The processed files were set as one experiment per each file to report the detected intensities in each of the processed files separately. Trypsin was set as the proteolytic enzyme and two missed cleavages were allowed. Cysteine carbamidomethylation was selected as a fixed modification. Oxidation of methionine and protein N-terminal acetylation were searched as variable protein modifications. Match between runs was switched off for samples of the same plant genus analyzed together. A Bruker Q-TOF instrument was selected and default tolerances were used (0.07 Da for the first search and 0.006 Da for the main peptide search at the MS level). Protein identification was performed using default 40 ppm as the mass tolerance at the MS/MS level for the TOF analyzer. The minimal required peptide length was set to seven amino acids. For each tested plant host, either reference proteome with all isoforms was downloaded from the UniProt protein knowledgebase [49] (accessed 3 January 2020) or, if not present, all available protein sequences for the given taxonomic plant genus from UniProt were used instead. For *Nicotiana benthamiana*, a homology-guided proteome [39] derived from *Nicotiana tabacum* was also included (see UniProt identifiers in Table 1). For each tested plant host, a host range property was searched in Plant Viruses Online [34] and Descriptions of Plant Viruses [35,36] online databases, and a list of potential virus pathogens for the tested plant genus was assembled. All available protein sequences for the obtained viruses were downloaded from the UniProt protein knowledgebase (accessed 3 January 2020, see also the PREPARATION section of Table 1) and their UniProt protein identifiers were supplied with the “UNIPROT_VIRUS_” prefix to easily identify detected virus proteins among the MaxQuant results. Thus, a protein sequence database for each tested host consisted of specific host proteins with original UniProt identifiers and virus proteins of potential virus pathogens for a selected host (with “UNIPROT_VIRUS_” prefix followed by their original UniProt identifier). The MaxQuant proteinGroups.txt and peptides.txt files were used for subsequent analyses of detected proteins and peptides in the samples and are included as Supplementary File S5.

### 4.4. Virus Protein Sequence Coverage, Virus Protein Putative Sequence

For each identified virus protein, all amino acid sequence isoforms, listed as separate entries in MaxQuant proteinGroup file, were examined. As a representative sequence for the isoform, the leading detected protein in each MaxQuant proteinGroup record was selected; if it was reported as a fragment in its fasta header, then other proteins of the protein group with the same or longer sequence and with the same number of detected fragments were considered. All the identified peptide fragments for the isoforms were aligned to the sequence using R Software 4.0 [50] and in-house scripts (for download, see http://uprt.vscht.cz/aaseq). The viral protein isoform sequence with the highest sequence coverage was then selected as a representative sequence for the identified virus protein at all. For these sequences with highest detected coverages, detected peptide fragments, their alignment, and supposed trypsin sites, see Supplementary File S2. For viruses expressing the polyprotein, the protein sequence, detected peptide fragments and their alignment are reported only if peptide fragments were also detected in other regions than in the capsid protein sequence; otherwise, only their capsid protein sequence is listed.

In MaxQuant software, minimal length for peptide detection was set to seven amino acids. Thus, for each reported protein sequence, a relative coverage was also computed for the sequence with all the peptide fragments shorter than seven amino acids omitted (see Table 1 and Supplementary File S2). The task was done using the trypsin digest sites detected according to the rules of ExPASy PeptideCutter online tool [50] and R Software 4.0 in-house scripts (for download, see http://uprt.vscht.cz/aaseq).

To construct a putative sequence maximizing the sequence coverage by detected peptide fragments, all the identified peptide fragments in the sample were extracted from MaxQuant search result files. For each examined viral protein, a protein sequence isoform with the highest sequence coverage was selected as its original sequence. All the local alignments of detected peptides to the protein sequence were then determined using R Bioconductor package ‘Biostrings’ (version 2.58). The alignments overlapping the sequence borders were removed. For each remaining peptide alignment, the number of modifications (substitutions, insertions, deletions, or improper alignments to trypsin digest sites) to the original sequence was determined and only peptide alignments with at most ⌊fragment length/7⌋ modifications were used. For the conflicting alignments of the peptides, those with the higher ratio of modifications per length were removed (but are also separately reported). The putative sequence was then constructed based on the original protein sequence and modifications detected in the aligned peptides. For constructed putative sequences, detected peptide fragments, their alignment, the peptides with conflicting alignments, and the putative sequence amino acid coverage, see Supplementary File S3. The used in-house scripts can be downloaded at http://uprt.vscht.cz/aaseq.

### 4.5. Identification of Strain-Specific Sequences

For the determination of strain-specific protein sequences (WDV, PPV) or distinct BYDV disease species protein sequences, all available complete genome nucleotide sequences were downloaded from the NCBI database [51] for virus strains and BYDV disease species occurring in the Czech Republic, and the sequences were aligned using the ClustalX 2.1 tool [52]. The sequences of open reading frames were then translated into an amino acid sequence and the amino acid sequences were aligned again in ClustalX 2.1. For each tested region and virus strain/pathogen species, the consensus amino acid sequence was determined and reported as a strain-specific sequence. If no consensus sequence was detected, then the consensus amino acid sequences for sequence subgroups were determined and used instead.

## Figures and Tables

**Table 1:** Identified host and virus proteins from single-leaf-tissue samples of virus inoculated plants with strong symptoms.

	**Sample**	01a/barley *^1^	01b/barley	01c/barley	02/wheat *^1^	03a/Chinese cabbage	03b/Chinese cabbage	04a/tobacco	04b/tobacco	04c/tobacco	04d/tobacco	04e/tobacco	05/apricot	06a/plum	06b/plum	07/bean	08/goosefoot	09/sorghum	10/maize
**PREPARATION**	**Sample weight (g)**	0.14	0.34	0.46	0.34	0.52	0.47	0.47	0.46	0.45	0.55	0.63	0.60	0.60	0.97	0.48	0.63	0.32	0.13
	**Host taxonomic classification**	*Hordeum* *vulgare*	*Hordeum* *vulgare*	*Hordeum* *vulgare*	*Triticum* *aestivum*	*Brassica rapa subsp. pekinensis*	*Brassica rapa subsp. pekinensis*	*Nicotiana benthamiana*	*Nicotiana benthamiana*	*Nicotiana benthamiana*	*Nicotiana clevelandia x N. glutinosa*	*Nicotiana* *tabacum*	*Prunus* *armeniaca*	*Prunus* *domestica*	*Prunus* *Pdomestica*	*Phaseolus* *vulgaris*	*Chenopodium amaranticolor*	*Sorghum* *bicolor*	*Zea mays*
	**UniProt reference proteome used**	UP000011116			UP000019116	UP000011750		UP000084051 *^2^					*^3^			UP000000226	*^4^	UP000000768	UP000007305
	**Reference genome proteins**	189,799			130,673	40,809		73,605 + 53,411 + 74,802 *^2^					155,017 *^3^			30,501	1063 *^4^	41,380	99,254
	**Known host viruses in online databases**	66			63	29		369					17			227	378	22	69
	**Tested viruses for sample (UniProt protein sequences available)**	47			45	24		232					13			151	232	18	52
**RESULTS**	**Identified peptides in sample**	3233	2871	3963	3888	1844	1094	3176	2772	3412	3426	1270	2344	1648	1047	4364	346	1942	1956
	Identified host proteins *	886	810	1082	1076	537	339	756	668	865	865	287	593	435	362	1163	71	570	549
	**Identified virus**	Wheat dwarf virus (WDV)	Barley yellow dwarf virus (BYDV)	Brome mosaic virus (BMV)	Wheat dwarf virus (WDV)	Cauliflower mosaic virus (CaMV)	Turnip vein clearing virus (TVCV)	Plum pox virus (PPV)	Turnip mosaic virus (TuMV)	Tobacco ringspot virus (TRSV)	Tomato aspermy virus (TAV)	Tobacco mosaic virus (TMV)	Plum pox virus (PPV)	Plum pox virus (PPV)	Plum pox virus (PPV)	Bean common mosaic virus (BCMV)	Broad Bean Wilt Virus 2 (BBWV-2)	Sorghum mosaic virus (SrMV)	Sugarcane mosaic virus (SCMV)
	**Identified virus family**	Geminiviridae (DNA)	Luteoviridae (RNA)	Bromoviridae (RNA)	Geminiviridae (DNA)	Caulimoviridae (DNA)	Virgaviridae (RNA)	Potyviridae (RNA)	Potyviridae (RNA)	Secoviridae (RNA)	Bromoviridae (RNA)	Virgaviridae (RNA)	Potyviridae (RNA)	Potyviridae (RNA)	Potyviridae (RNA)	Potyviridae (RNA)	Secoviridae (RNA)	Potyviridae (RNA)	Potyviridae (RNA)
	Identified capsid protein peptides **	10	2	11	10	10	6	11	14	8	12	12	6	6	5	4	17	9	4
	Capsid protein sequence coverage (AA) **	147 of 260	36 of 199	147 of 189	147 of 260	110 of 489	82 of 157	119 of 330	154 of 286	104 of 513	134 of 218	92 of 159	86 of 330	65 of 330	57 of 330	71 of 287	247 of 599	95 of 320	59 of 313
		(56.5%)	(18.1%)	(77.8%)	(56.5%)	(22.5%)	(52.2%)	(36.1%)	(53.8%)	(20.3%)	(61.5%)	(57.9%)	(26.0%)	(19.7%)	(17.2%)	(24.7%)	(41.2%)	(29.7%)	(18.8%)
	Capsid protein sequence coverage on ≥ 7 amino acid sequences (AA) **	147 of 189	36 of 148	147 of 152	147 of 189	110 of 333	82 of 142	119 of 278	154 of 210	104 of 437	134 of 187	92 of 148	86 of 278	65 of 278	57 of 278	71 of 232	247 of 506	95 of 211	59 of 247
		(77.8%)	(24.3%)	(96.7%)	(77.8%)	(33.0%)	(57.7%)	(42.8%)	(73.3%)	(23.8%)	(71.7%)	(62.2%)	(30.1%)	(23.4%)	(20.5%)	(30.6%)	(48.8%)	(45.0%)	(23.9%)
	Other viral proteins identified **	−	2 fragments of movement protein	−	−	more than 10 fragments of other viral proteins - movement protein, reverse transcriptase, aphid transmission protein, etc.	−	other more than 10 fragments of genome polyprotein	other more than 10 fragments of genome polyprotein	−	−	more than 10 fragments of replication protein	other more than 10 fragments of genome polyprotein	other 6 fragments of genome polyprotein	−	other more than 10 fragments of genome polyprotein	one other fragment of genome polyprotein from RNA 1 component	other more than 10 fragments of genome polyprotein	other more than 10 fragments of genome polyprotein
	Sequence covered (aa) **	147	87	147	147	794	82	936	695	104	134	405	291	126	57	380	275	486	343
	**Putative sequence** covered (aa) ***	147	87	147	147	807	82	1073	734	104	142	405	291	126	57	407	327	522	398
	Virus presence also confirmed by ****	DAS-ELISA qPCR	DAS-ELISA RT-PCR RFLP	DAS-ELISA	DAS-ELISA qPCR	DAS-ELISA	DAS-ELISA	DAS-ELISA qPCR	DAS-ELISA qPCR	DAS-ELISA	DAS-ELISA	DAS-ELISA	DAS-ELISA qPCR	DAS-ELISA qPCR	DAS-ELISA qPCR	DAS-ELISA	electron microscopy RT-PCR	DAS-ELISA	DAS-ELISA

* total count of identified MaxQuant proteinGroups containing only host proteins, without contaminants, proteins from reverse database, and without proteins only identified by site (by their peptide mass only, without supporting MS/MS spectrum); ** see also Supplementary File S2; *** see also Supplementary File S3 **** see Supplementary File S4 for method details; *^1^ used extraction protocol without RuBisCO depletion step; *^2^ tested against UP000084051 reference proteome of *Nicotiana tabacum* (73,605 proteins) together with assembled proteome for *Nicotiana benthamiana* (ref. [39], NbD_AA—53,411 proteins, NbE_AA—74,802 proteins); *^3^ no reference proteome is available in UniProt database, and thus, tested against all available protein sequences of *Prunus* genus; *^4^ no reference proteome is available in UniProt database, and thus, tested against all available protein sequences of *Chenopodium* genus.

## Supplementary Materials

The following are available online at https://www.mdpi.com/2076-0817/9/11/966/s1. Table S1: Taxonomic classification and brief description of tested plant virus pathogens; Figure S1: Mechanical roller steel-ball crusher homogenizer (Homogenizer Hand Model, Art. No. 400010, Bioreba, Switzerland) for proper tissue homogenization; File S1: Plant cultivation conditions and virus inoculation for tested hosts; File S2: Viral protein sequences, detected peptide fragments, their alignment, and amino acid sequence coverage—isolate sequences with most detected peptides); File S3: Putative viral protein sequences maximizing the sequence coverage, together with detected peptide fragments. The sequences are based on identified protein database sequence with the highest coverage, followed by the inclusion of other detected peptides with only a few modifications to the original sequence (see the Materials and Methods Section 4.4); File S4: Other Biochemical methods used for confirmation of detected viral pathogens; File S5: The MaxQuant proteinGroups.txt and peptides.txt files from virus inoculated samples used for subsequent analyses of detected proteins and peptides. References [53,54,55,56,57,58,59,60,61,62,63,64,65,66,67,68,69,70,71,72,73,74,75,76,77,78,79,80,81,82,83,84,85,86] are cited in the supplementary materials.
