# Supplementary material for: Efficient Confirmation of Plant Viral Proteins and Identification of Specific Viral Strains by nanoLC-ESI-Q-TOF Using Single-Leaf-Tissue Samples
Source: Pathogens. 2020 Nov 19;9(11):966. doi: 10.3390/pathogens9110966 (PMC7699591; doi:10.3390/pathogens9110966)
Supplement: Supplementary file 1 [file pathogens-09-00966-s001.zip › Supplementary file 1 - XML.docx]

# **Plant cultivation conditions and virus inoculation**

For mechanical inoculation, a distilled-water-based phosphate buffer was added to the infected material in the ratio of 3:1 (v/w), and carborundum powder (Carborundum Electrite, Czech Republic) was also supplemented. The homogenate was rubbed with fingers (in latex gloves) or a sponge onto young leaves of inoculated plants. After inoculation, the plants were sprayed with distilled water and grown in a greenhouse (20-22 °C cultivation temperature, 14 h/10 h day/night regime). In this way, the TRSV inoculation (0.02 M phosphate buffer, pH 7.0) of approximately one month old *Nicotiana benthamiana* was applied, and the TAV inoculation (0.066 M phosphate buffer, pH 7.0) of approximately one month old *Nicotiana clevelandia x glutinosa* was applied. The TMV inoculation (0.05 M phosphate buffer, pH 7.0, inoculum maintained at the Dept. of Plant Protection, Czech University of Life Sciences, Prague) of approximately one month old *Nicotiana tabacum* (three leaves) was applied, and the plants were left for more than another month to develop strong symptoms. Similarly, the CaMV inoculation (0.066 M phosphate buffer, pH 7.0) of a *Brassica rapa subsp. pekinensis* plant with three or four leaves was applied and left for another two months (20 °C/12 °C 14 h/10 h day/night regime). The TVCV inoculation (0.05 M phosphate buffer, pH 7.0, inoculum originally from wild garlic mustard (*Alliaria petiolata*)) of *Brassica rapa subsp. pekinensis* plant with one true leaf was applied and left for another two months to develop strong symptoms. The BBWV-2 inoculation (0.07 M phosphate buffer, pH 7.0, inoculum originally from field-planted peppers [79]) of a few weeks old plant was applied (left 24 h before inoculation in dark) and left for approximately month (25 °C/20 °C 14 h/10 h day/night regime) before collecting the sample. The SCMV and SrMV inoculation (both 0.05 M phosphate buffer, pH 7.0, inoculum purchased from Leibnitz Institute – DSMZ, Germany) of maize and sorghum with one true leaf was applied, and the plants were left for more than another month to develop strong symptoms. The BMV inoculation (0.02 M phosphate buffer, pH 7.0) of two or three leaves of plants of *Hordeum vulgare* winter barley cv. Fabian was applied and left for more than another month to develop strong symptoms.

For BCMV, the isolate used in the work was maintained in plants of the common bean, (*Phaseolus vulgaris L.*) cv. Saxa, known for a high level of seed transmissibility of BCMV, even for long-term maintaining of the virus. The plants were grown in the experimental plot of the Crop Research Institute, Prague, Czech Republic. From infected symptomatic plants, seeds were collected and used for raising the new plant carrying BCMV and sampled after approximately 6 weeks.

PPV-D and PPV-Rec used isolates [80] that are maintained in their natural hosts (plum and apricot trees) grown in the screenhouse of the Crop Research Institute, Prague, Czech Republic. The plum and apricot trees were originally inoculated by grafting, and the sampled trees were several years old.

The PPV CAH-2 belonging to the PPV-M strain [80] and TuMV isolates were transmitted to *Nicotiana benthamiana* plants by mechanical inoculation. The viral inoculum was prepared by homogenizing leaves from systemically infected leaves 1:10 (w/v) in 0.1 M phosphate buffer, pH 7.2. The inoculated plants were grown in an insect-proof cultivation room under controlled conditions (22 °C/18 ^o^C 14 h/10 h day/night regime). The experimental plants infected by PPV and TuMV were sampled approximately one month after inoculation; they displayed pronounced symptoms as soon as two weeks after inoculation.

For WDV and BYDV, the inoculation was administered similarly to the process described by Viktorova*, et al.* [81]. For BYDV, many viruliferous aphids, *Rhopalosiphum padi,* carrying the BYDV-PAV virus isolate were transmitted to two or three leaves of a spring barley plant, cv. Sebastian, and left there for several days. After that, the plant was treated with insecticide (Mospilan, acetamiprid active ingredient) and kept under controlled conditions (temperature 19/15˚C, 16 h/8 h day/night) for another two months to develop strong symptoms. For WDV, the viruliferous leafhoppers *Psammotettix alienus* carrying the WDV-B strain were transmitted to *Hordeum vulgare* winter barley cv. Doreen and maintained there several days; after that, the leafhoppers were removed by hand. The plants were then kept under controlled conditions (temperature 19/15 ˚C, 16 h/8 h day/night) for the next two months. Similarly, viruliferous leafhoppers, *Psammotettix alienus,* carrying the WDV-W strain were applied to *Triticum aestivum* spring wheat, cv. Fielder.
