# Supplementary material for: Efficient Confirmation of Plant Viral Proteins and Identification of Specific Viral Strains by nanoLC-ESI-Q-TOF Using Single-Leaf-Tissue Samples
Source: Pathogens. 2020 Nov 19;9(11):966. doi: 10.3390/pathogens9110966 (PMC7699591; doi:10.3390/pathogens9110966)
Supplement: Supplementary file 1 [file pathogens-09-00966-s001.zip › Supplementary file 4 - XML.docx]

# **Confirmation of detected viral pathogens by other methods**

The successful inoculation of most viral pathogens in sampled tissues was confirmed by DAS-ELISA [82] according to manufacturer’s protocol (Bioreba, Switzerland: PPV, TuMV; Loewe Biochemica, Germany: BBWV-2, BCMV, SCMV, SrMV; Sediag, France: BMV, BYDV, CaMV, TAV, TRSV, WDV; Leibnitz Institute DSMZ, Germany: TVCV, TMV, SCMV, SrMV).

For PPV and TuMV in tobacco, to complement the serological test, a two-step RT-PCR was also carried out using virus-specific primers. Total RNAs were extracted from symptomatic leaves using the NucleoSpin RNA Plant kit (Macherey-Nagel). The first-strand cDNA was synthesized by reverse transcription of total RNA using random hexamer pdN6 primers and the Avian myeloblastosis virus (AMV) reverse transcriptase (both Promega, U.S.A.). An aliquot of cDNA was used subsequently in PCR performed with GoTaq G2 Green Master Mix (Promega) with specific primers (for PPV, PPV-NCuniFor, 5´-GAGGCAATTTGTGCTTCAATGG-3´, and PPV-NCuniRev, 5´-CGCTTAACTCCTTCATACCAAG-3´, Predajna, *et al.* [83], for TuMV, TuMV 8209F, 5´-ATTCTTCGTCAATGGAGACG-3´, and TuMV 8903R, 5´-AAGTTCCAGAAGTTCCAGCG-3´, (Glasa, unpublished). For both primer combinations initial denaturation was done at 94 °C for 1 min followed by 35 cycles of 94 °C for 30 s, 56 °C for 35 s, and 72 °C for 1 min and the final extension step at 72 °C for 10 min.

The presence of BBWV-2 in the inoculated goosefoot plants was proven by electron microscopy and RT-PCR. Under electron microscopy, isometric particles of about 25 nm corresponding in size and shape to the described particles of BBWV-2 [53] were observed. For RT-PCR, the total RNA was extracted from the inoculated plants. PCRs with primer pairs specific to four virus genome regions [84] were carried out after reverse transcription to cDNA using oligo-dT and random hexamers. Amplicons of expected sizes (for Fab5´R1 ~400 bp; for Pol2 ~460 bp; for MP4 ~500 bp and for SCP ~250 bp) were obtained in all reactions.

For WDV, the pathogen was also detected by qPCR [82]. Strain-specific discrimination was done using the qPCR TaqMan assay [85]. For PPV, strain differentiation was done by strain-specific RT-PCR assay according to Subr*, et al.* [86]. The BYDV disease species discrimination was confirmed by RT-PCR and restriction fragment length polymorphism (RFLP) as in Kundu*, et al.* [40].
