# Supplementary material for: Efficient Confirmation of Plant Viral Proteins and Identification of Specific Viral Strains by nanoLC-ESI-Q-TOF Using Single-Leaf-Tissue Samples
Source: Pathogens. 2020 Nov 19;9(11):966. doi: 10.3390/pathogens9110966 (PMC7699591; doi:10.3390/pathogens9110966)
Supplement: Supplementary file 1 [file pathogens-09-00966-s001.zip › Supplementary Table ST1 - XML.docx]

**Supplementary Table ST1.** Taxonomic classification and brief description of tested plant virus pathogens.

| BBWV-2 (genus *Fabavirus)* is an economically important virus that probably occurs worldwide [53]. The virus is easily spread mechanically and in a non-persistent manner by aphids to many host plant species. The most common host species are broad bean (*Vicia faba*), pepper (*Capsicum frutescens*), pea (*Pisum sativum*) and sugar beet (*Beta vulgaris*) where it causes systemic mosaic on leaves and loses in the crop yield [54]. |
| --- |
| BMV (genus *Bromovirus*) is an excellent model for a virus with a multipartite RNA genome [55]. Also, it is often used as a vector for virus-induced gene silencing [56]. It infects many cereal crops and wild grasses, but also dicotyledonous plants. The virus is widely distributed globally and considered to be primarily mechanically transmitted. However, it is not thought to cause significant economic losses [57]. |
| BYDV disease is one of the most important cereal virus diseases worldwide. It causes significant yield losses [58] with host range of almost the whole *Poaceae* family. The disease is caused by at least eight different species of genera *Luteovirus* (BYDV-PAV, BYDV-PAS, BYDV-MAV a BYDV-GAV), *Polerovirus* (CYDV-RPV), or without genus classification (BYDV-RMV, BYDV-SGV, BYDV-GPV) [59], and it is transmitted by several aphid species. BYDV-PAV is probably the most widespread cereal virus in the world [60]. In the Czech Republic, three different distinct BYDV species were detected, BYDV – PAV, PAS, and MAV [40]. |
| CaMV (genus *Caulimovirus*) is a DNA virus spread worldwide, especially in vegetable crops. It was the ﬁrst plant virus to be discovered to contain DNA instead of RNA [61]. CaMV is transmitted by aphids in a non-circulative manner, semi-persistently, most often by *Myzus persicae* [62,63]. The host range of CaMV is limited to plant species of the *Brassicaceae* and *Solanaceae* families. |
| PPV (genus *Potyvirus*) is the agent responsible for the Sharka disease, the most important viral disease of cultivated ornamental or wild Prunus trees. PPV poses a serious threat to stone fruit cultivation worldwide. To date, at least 10 genetically different strains of PPV have been identified, from which PPV-M, PPV-D, and their recombination event strain, PPV-Rec, have global epidemiological impact [64]. |
| SCMV and SrMV (both genus *Potyvirus*) are transmitted mechanically and also non-persistently by several species of aphids infecting plants from Poaceae family. They can have a high impact on infected sugarcane and maize or sorghum [65,66]. Both viruses cause leaf mottling, reddening, and/or stunting of plants and are spread worldwide. |
| TAV (genus *Cucumovirus*) is an important viral pathogen of chrysanthemums [67], but it also has a wide range of hosts. The virus is readily transmitted by several species of aphids in a non-persistent manner and by inoculation of sap. It is widespread and common in countries cultivating chrysanthemums and relatively uncommon in tomatoes [36]. |
| TMV (genus *Tobamovirus*) was the first described plant infecting virus [68]. It is an important pathogen of some crops like tobacco, tomatoes, and peppers. Thanks to its easy transmissibility and stability of particles, it is frequently used as a model for various studies of plant viruses. |
| TRSV (genus *Nepovirus*) has a wide host range including fruit trees, ornamentals, and various weeds. It occurs in both an­nual and perennial crops. It is readily transmissible by sap inocu­lation and naturally transmitted by nematodes of selected *Xiphinema* spp*.*, by pollen, and also by seeds of selected crops and weed hosts [69]. TRSV is a quarantine pathogen in Europe [70]. |
| TuMV (genus *Potyvirus*) is spread worldwide and infects a wide range of plants, primarily dicotyledonous cruciferous plant species including vegetables, oilseeds, forage crops, ornamentals or weeds. TuMV is transmitted by aphids in a non-persistent manner [71,72]. |
| TVCV (genus *Tobamovirus*) was first characterized in Oklahoma, U.S.A. in 1993. It has been mostly isolated from cruciferous host plants. However, it also infects tobacco and other plants and sometimes it was confused with TMV. On crucifers it usually causes vein clearing, but often the infection is symptomless. Tobacco reacts to infection by TVCV similar to the infection by TMV [73]. |
| WDV (genus *Mastrevirus*) is one of the most important cereal viral pathogens in Europe, especially in countries cultivating winter crops [74,75], but it has also been found outside of Europe. This DNA virus is transmitted by the leafhopper *Psammotettix alienus* in a circulative, non-propagative manner [76,77]. Two main strains are known, the wheat-adapted strain (WDV-W) and the barley-adapted strain (WDV-B), with an overlapping host range [77]. However, a more detailed classification of strains also exists [78]. |
