# Supplementary figures and images for: Efficient Confirmation of Plant Viral Proteins and Identification of Specific Viral Strains by nanoLC-ESI-Q-TOF Using Single-Leaf-Tissue Samples
Source: Pathogens. 2020 Nov 19;9(11):966. doi: 10.3390/pathogens9110966 (PMC7699591; doi:10.3390/pathogens9110966)

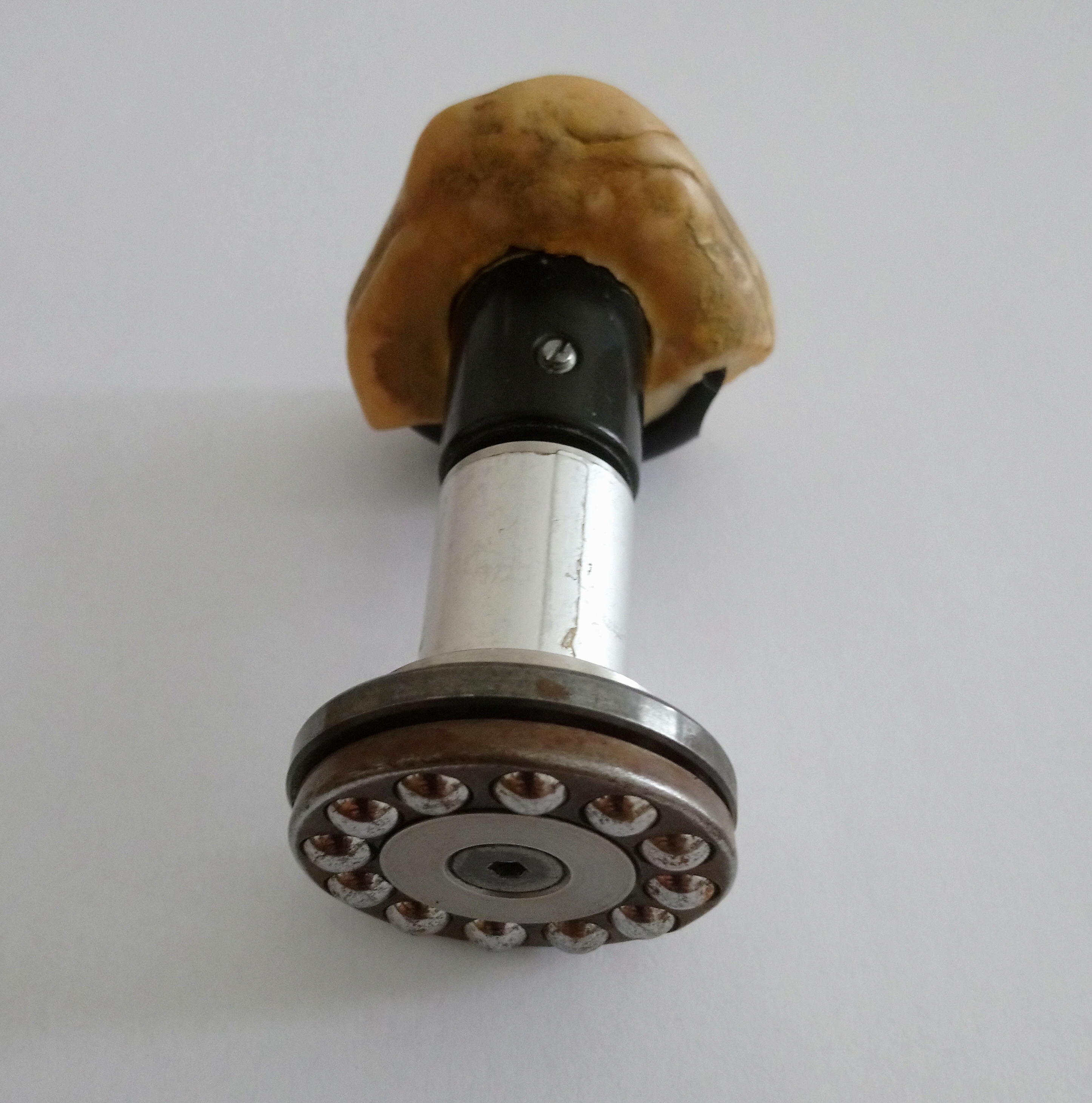

Supplement: Supplementary file 1 [file pathogens-09-00966-s001.zip › Supplementary Figure SF1.jpg]
